# Barrier-Guided Local Nanopharmaceutics for Cartilage and Osteochondral Regeneration: From Effective Tissue Exposure to Cell-State Control and Translational Quality Attributes

**DOI:** 10.3390/pharmaceutics18101206

**Published:** 2026-09-23

**Authors:** Tao Shen, Junxu Zhu, Zongyan Cai, Wenhao Guo, Jiaxin Jin

**Affiliations:** Department of Orthopedics, The Second Hospital of Lanzhou University, The Second Clinical Medical School, Gansu Provincial Bone and Joint Research Institute, Lanzhou University, Lanzhou 730030, China

**Keywords:** local nanopharmaceutics, nanomaterials, cartilage regeneration, osteochondral regeneration, intra-articular drug delivery, nanocarriers, nanocomposite hydrogels, extracellular vesicles, cell-state control, critical quality attributes

## Abstract

Local nanopharmaceutic systems are increasingly used to prolong intra-articular residence, improve cartilage access, control local release, and regulate repair-relevant cell states. However, joint retention is often mistaken for effective tissue exposure, and nanoscale complexity is frequently treated as an advantage without demonstrating which transport, biological, or mechanical barrier it resolves. This narrative review develops a barrier-guided framework for nanocarriers, nano-enabled hydrogels, extracellular vesicles, and spatial osteochondral constructs. We distinguish nominal formulation identity from the delivered material state that emerges after dilution, protein adsorption, mechanical loading, and degradation, and we separate joint-cavity persistence, cartilage partitioning and penetration, and bioavailable cargo at target cells. The evidence is strongest when a controllable material attribute is linked to a depth-resolved exposure or repair function; it remains uneven for coupled loading–release behavior, direct cross-platform comparisons, and transferable acceptance limits. We therefore distinguish published quantitative anchors from product-specific specifications and propose a staged workflow that defines two-sided failure boundaries for size and dispersity, binding–dissociation kinetics, stress relaxation, dose, and potency. Across platforms, the practical priorities are reversible rather than maximal binding, matched static-versus-loaded release testing, mechanism-necessity controls for extracellular vesicles, modality-aware fate measurements, and critical quality attributes linked to in vivo performance. The most credible strategy is the minimum necessary complexity that preserves a measurable structure–property–exposure–function relationship from manufacture through remodeling.

## 1. Introduction: From Nano-Enabled Materials to Local Nanopharmaceutics

Cartilage and osteochondral repair remain difficult because the target is not an isolated defect but a mechanically loaded, avascular, matrix-dense, and immunologically active joint. Mature articular cartilage contains few cells and a highly organized extracellular matrix (ECM) that restricts solute transport, cell migration, and matrix restoration after injury [1,2,3]. Osteochondral lesions add a second design problem: cartilage, calcified cartilage, and subchondral bone require different mechanical, biochemical, and vascular conditions [4,5]. Accordingly, scaffold architecture, local delivery, and host response must be interpreted as coupled determinants of repair rather than independent material features [6,7,8].

Established clinical strategies illustrate why defect filling and regeneration are not equivalent. Microfracture recruits marrow-derived cells, but repair tissue commonly exhibits fibrocartilaginous features and may deteriorate with time [9,10,11]. Autologous chondrocyte implantation can improve selected focal defects, yet donor-site morbidity, reoperation, hypertrophy, and variable integration remain important limitations [12,13,14]. These outcomes define a stringent benchmark for nano-enabled strategies: transient biochemical improvement is insufficient unless it produces stable matrix composition, load transfer, and integration.

These limitations shift the design problem from adding a novel material to controlling a sequence of material–tissue interactions. Classical tissue engineering integrates cells, scaffolds, and signals, but each added function can also introduce variability or a new failure mode [15,16]. In this review, a platform is considered functionally nano-enabled only when its nanoscale feature changes a rate-limiting process—such as cartilage partitioning, matrix penetration, payload release, cell uptake, protein adsorption, or mechanotransduction—in a way that can be measured against a non-nano comparator [17,18]. Nanoscale classification alone is therefore not evidence of therapeutic advantage.

We use local nanopharmaceutics to mean the formulation science of controlling the delivered state and active exposure of nano-enabled systems within the joint, rather than a new material class. The central pharmaceutic problem is effective exposure. Intra-articular administration is anatomically local, but it does not ensure that an active payload reaches cartilage, remains bioavailable, or acts for the required duration. Cartilage-delivery studies distinguish joint-cavity persistence from tissue partitioning and depth-dependent transport [19,20], while translational analyses show that nominal composition may not be preserved after manufacturing, storage, protein adsorption, or degradation [21,22]. We therefore distinguish the prepared formulation from its delivered material state in the joint.

We propose a barrier-guided development sequence: identify the dominant loss of exposure or function; select the minimum material attribute expected to correct it; measure the resulting tissue interaction; and test the competing failure mode. The hierarchy is dynamic rather than universal. Early inflammatory lesions may be limited by clearance and synovial uptake, whereas intact but dense cartilage may be limited by penetration; full-thickness defects may instead be dominated by mechanical localization and zonal integration. Charge-mediated systems make this logic explicit because stronger binding can improve retention while worsening surface sequestration [23,24].

## 2. Review Scope and Evidence Synthesis

### 2.1. Scope: Regeneration Versus Disease-Modifying Delivery

This review focuses on nanomaterials for cartilage and osteochondral regeneration rather than symptomatic osteoarthritis treatment alone. Focal defects, traumatic osteochondral lesions, and degenerative joint states overlap clinically but do not present the same delivery or repair barriers [4,5,25]. Osteoarthritis evidence is therefore included only when it clarifies cartilage transport, synovial interactions, inflammatory and senescent cell states, or translational constraints directly relevant to structural repair [1,26,27].

This distinction is essential for claim discipline. Prolonged residence, cytokine reduction, or protection against matrix loss can demonstrate local pharmacology, but not necessarily regeneration. Regenerative claims require evidence of stable cartilage phenotype, appropriate matrix composition, osteochondral integration, mechanical competence, and durable function. A nanocarrier that suppresses synovitis and a construct that rebuilds a full-thickness osteochondral defect may share materials, yet they solve different problems [4,28,29]. Joint-as-organ and disease-primer literature defines the pathological context [26,30,31], while cartilage and subchondral-matrix studies define the tissue barriers against which structural repair must be judged [32,33,34].

Previous reviews have clarified nanoparticle–cartilage interactions and intra-articular delivery [19,20,35], controlled-release hydrogels and extracellular-vesicle standards [36,37], and three-dimensional osteochondral printing [38,39,40]. These contributions are commonly organized by material class or fabrication route. The distinct contribution here is to connect nanoscale attributes to a causal sequence—delivered material state, effective tissue exposure, cell-state response, and repair function—and identify the test that could falsify each proposed advantage.

### 2.2. Literature Search and Evidence Selection

This narrative review was informed by iterative, barrier-focused searches of PubMed and publisher databases, with the final update performed on 31 July 2026, and by backward and forward citation tracing from foundational studies and recent reviews. Search blocks paired cartilage or osteochondral regeneration with nanomaterial, nanoparticle, nanocarrier, hydrogel, extracellular vesicle, protein corona, mechanobiology, viscoelasticity, stress relaxation, macrophage, tribology, spatial scaffold, interface mechanics, large-animal model, degradation, in vivo fate, or critical quality attribute. Because terms were refined iteratively to test mechanism-specific questions, a single PRISMA-style screening denominator is not claimed. The 148-source total refers to papers, standards, and guidance retained in the synthesis, not to the number of records screened. Primary studies were prioritized for causal claims; standards, regulatory guidance, and reviews were used for terminology, reporting expectations, and evidence context. The objective was mechanistic integration rather than quantitative effect estimation; therefore, no formal meta-analysis or risk-of-bias score was applied.

Evidence was considered in three tiers. Direct cartilage or osteochondral nanomaterial studies supported tissue-specific claims; joint-delivery and osteoarthritis studies were included when they directly informed synovial interaction, cartilage access, or local exposure; and general biomaterials or mechanobiology studies were used only for transferable structure–property principles. Records were not used as decisive mechanistic evidence when they addressed symptomatic osteoarthritis without structural-repair or delivery relevance; reported only bulk composition or viability without a measurable transport, loading, tissue, or interface outcome; lacked sufficient formulation, dose, or loading detail to interpret the claimed mechanism; or relied on a secondary account when the primary study was available. Duplicate reports and preprints superseded by peer-reviewed articles were also excluded from decisive claims. Priority was given to studies that isolated a material variable, connected it to a measurable tissue interaction, and reported mechanical, spatial, or in vivo outcomes. The qualitative evidence map used in this review is descriptive: it reflects apparent directness and convergence of cited evidence, not pooled effect sizes, formal certainty grades, or cross-platform rankings.

During manuscript preparation, generative tools were used only to assist with manuscript organization, English-language editing, and the drafting of non-data schematic elements. Literature retrieval, evidence selection, citation verification, scientific interpretation, and all final revisions were performed and checked by the authors. No generative tool was used to generate, alter, or analyze primary research data.

### 2.3. Analytical Framework: Formulation Identity, Delivered State, and Effective Exposure

We propose that nano-enabled repair be evaluated through five linked elements: a critical material attribute; the delivered material state produced by dilution, adsorption, loading, and degradation; effective exposure at the intended tissue and cells; the resulting cell-state or matrix response; and functional repair. A failure at any link invalidates inference from the preceding one. High loading efficiency, for example, does not establish cartilage exposure, and cartilage exposure does not establish stable regeneration.

This framework also defines a functional threshold for the term nanomaterial. A nanoscale component is mechanistically relevant only when removing or changing it alters a prespecified barrier-related function. This criterion separates causal nano-enablement from decorative nanoscale labeling and creates a direct comparator for platform selection [17,18,19,20,21,22,23,24,36].

Effective exposure should be resolved at three levels: persistence in the joint cavity; partitioning and penetration into cartilage or the defect; and bioavailable cargo at target cells. These levels are coupled but not interchangeable. Their separation provides a common analytical language for synthetic nanocarriers, EVs, nano-enabled hydrogels, and spatial constructs and exposes the trade-offs between retention, penetration, release, and safety [17,18,19,20,21,22,23,24].

Figure 1 summarizes the barrier-guided framework by linking repair constraints to controllable material attributes, biological responses, spatial repair, and translational validation. Table 1 positions this analysis relative to adjacent reviews and emphasizes pharmaceutical questions about effective exposure, delivered material state, competing objectives, and mechanism-linked failure testing.

## 3. Biological, Transport, and Mechanical Barriers That Determine Nanomaterial Performance

### 3.1. Local Pharmacokinetic Barriers: Joint Residence, Cartilage Partitioning, and Cellular Access

Intra-articular administration initiates a local pharmacokinetic cascade rather than a single exposure event. A formulation must first persist in the joint cavity, then partition into the intended tissue, penetrate to an appropriate depth, release active cargo, and, finally, reach relevant cells. Dilution by synovial fluid, joint motion, vascular absorption, and lymphatic clearance can interrupt this cascade [28,29,41]. Particle and depot studies further show that prolonged cavity residence is not equivalent to cartilage exposure [42,43]. We therefore recommend reporting cavity, tissue, and cellular exposure as separate quantities.

Cartilage penetration adds a distinct material–matrix constraint. The collagen–proteoglycan network restricts transport according to hydrodynamic size, shape, charge, deformability, matrix affinity, and disease-dependent loss of glycosaminoglycans [33,34]. Rothenfluh et al. demonstrated that surface chemistry can enhance cartilage targeting and retention [17], whereas Geiger et al. showed that a cartilage-penetrating carrier improved the distribution and efficacy of a growth factor [18]. Together, these studies establish that retention and penetration are related but non-equivalent material functions.

Charge-mediated transport illustrates why nanomaterial properties must be interpreted kinetically and in a disease-dependent matrix. Electrostatic interactions with negatively charged cartilage can increase uptake [23,44], and multi-arm avidin constructs can tune loading, release, and joint kinetics [24,45]. However, glycosaminoglycan loss changes fixed-charge density, while high multivalency can produce superficial trapping. The relevant target is a binding–dissociation window that supports reversible partitioning and diffusion, not the highest possible affinity.

Temporal exposure is mechanically conditioned. Early inflammation may require prompt or pulsatile antioxidant and immunomodulatory activity, whereas matrix formation may require sustained chondrogenic signaling and later mechanical maturation. Reactive-oxygen-species-, pH-, or enzyme-responsive materials can couple release to pathology [36,46,47,48], but joint loading can alter depot deformation, fluid exchange, and release independently of the intended trigger. We use mechanopharmaceutics to denote the testable coupling among the applied loading waveform, formulation state, transport, and active-payload exposure. Direct cartilage-relevant studies that co-measure these elements remain sparse; mechanobiological response to loading alone is not evidence that a formulation preserves release. Accordingly, mechanopharmaceutical claims require matched static and loaded conditions together with concurrent measurements of material recovery and active cargo [49].

### 3.2. Disease-Dependent Nano–Bio Interfaces and Cell-State Instability

Regenerative failure is also a problem of unstable cell states. After cartilage injury, matrix fragments, abnormal loading, and inflammatory mediators activate chondrocytes, synovial cells, macrophages, and recruited progenitors [27,50,51]. Synovitis and senescence studies show that inflammation and aging-related programs can alter the repair environment before a formulation reaches its target [52,53,54]. Resident and inflammatory synovial macrophage subsets further indicate that joint inflammation is organized by spatially defined cell states rather than by a single cytokine axis [55,56].

Inflammation should not be reduced to an on/off target or a carrier-independent cargo effect. Material chemistry, degradation products, stiffness, topography, and particulate uptake can redirect synovial and macrophage states, although an early inflammatory phase remains necessary for debris clearance. Resident macrophages can maintain a protective synovial barrier, whereas other subsets sustain synovitis [55,56]. Material-directed immunomodulation should therefore be evaluated as a time-resolved transition toward resolution and tissue support rather than a binary pro-inflammatory/reparative label. Blank carriers and unloaded matrices are needed to separate material from payload effects in systems intended to reprogram macrophage–chondrocyte communication [57,58,59,60,61].

Cell-state stabilization is equally important. Defect-recruited mesenchymal stromal cells do not inevitably form stable hyaline cartilage; their fate depends on matrix mechanics, oxygen, inflammation, ionic cues, ligand presentation, and soluble signals. Increased SRY-box transcription factor 9 (SOX9), collagen type II alpha 1 chain (COL2A1), or aggrecan (ACAN) is insufficient if collagen type I alpha 1 chain (COL1A1), collagen type X alpha 1 chain (COL10A1), runt-related transcription factor 2 (RUNX2), or matrix metallopeptidase 13 (MMP13) is co-induced. Single-cell studies identify fibrotic, hypertrophic, and catabolic trajectories [62,63,64], while instructive matrices show that biomaterial cues can redirect lineage and phenotype [65,66].

These observations shift biomaterial evaluation from cell survival to cell-state control. Relevant endpoints should determine whether a material stabilizes repair-compatible chondrocytes and progenitors, supports immune resolution, and avoids fibrotic, hypertrophic, or aberrantly mineralized states. Viability and gross histology remain necessary, but they are insufficient to establish a mechanistically appropriate host response.

Mechanosensitive signaling links material properties to cell state. Matrix stiffness, stress relaxation, and dynamic loading alter integrin–cytoskeletal tension, Hippo-pathway activity, and ion-channel signaling. In chondrocytes, transient receptor potential vanilloid 4 (TRPV4) and piezo-type mechanosensitive ion channel component 1 (PIEZO1) produce distinct transcriptional responses; similar mechanical inputs may support homeostasis or amplify injury depending on magnitude, duration, and matrix context [67,68]. Biomaterial studies should report both bulk modulus and the temporal and spatial signals transmitted to cells.

### 3.3. Zonal, Interfacial, and Material-State Barriers

Osteochondral repair adds a spatial and mechanical barrier that is absent from isolated cartilage resurfacing. The cartilage layer requires a non-mineralized, proteoglycan-rich, and low-vascular environment, whereas subchondral bone requires mineralization, vascular ingrowth, and load-bearing support. Calcified cartilage provides a transitional region for tissue compartmentalization and load transfer [4,5,69]. Biomaterials must therefore deliver distinct—and sometimes antagonistic—biochemical and mechanical cues at submillimeter-to-millimeter resolution while preventing cross-layer leakage.

Layered scaffolds, gradient hydrogels, and three-dimensional printed constructs can create regional architecture, but spatial appearance does not guarantee spatial function. Gradient and multilayered studies show how regional composition can be fabricated [40,69,70,71,72], whereas bioprinting and woven constructs extend this concept toward mechanically relevant, multicellular architectures [39,73,74,75]. A recent continuously graded hydrogel illustrates how spatially programmed mechanics and cues can be coupled within one construct [72], while scaffold-guided repair data indicate that failure to restore subchondral support can compromise the overlying cartilage despite a chondrogenic component [76]. These studies strengthen the case for coupled regional design but do not establish the general superiority of one material class. Failure can still occur through cross-layer diffusion, delamination, discontinuous load transfer, mismatched degradation, or exposure of cartilage to osteogenic cues. Spatial biomaterials therefore require simultaneous control of regional signaling, interface mechanics, and tissue replacement.

A further barrier is loss of functional material identity between manufacture and in vivo remodeling. Sterilization, storage, injection, protein adsorption, or cyclic loading can alter particle size, surface chemistry, ligand accessibility, network structure, filler dispersion, and degradation [21,22,77,78]. We call this in-joint configuration the delivered material state. If it changes, cartilage access, cell response, and interface performance may change despite an identical nominal composition. Figure 2 operationalizes these barriers as an integration-oriented design problem, and Table 2 links each barrier to a decisive validation test.

## 4. Local Nanopharmaceutic Platforms: Nanocarriers and Nano-Enabled Hydrogels

### 4.1. Physicochemical Control of Effective Exposure: Size, Charge, Affinity, and Release

Nanocarriers should be evaluated as regulators of a multistep exposure pathway rather than passive drug containers. Size, shape, deformability, charge, ligand density, matrix affinity, and protein adsorption jointly determine cavity persistence, cartilage partitioning, depth-dependent transport, and cell uptake. Foundational studies show that retention and penetration can be engineered independently [17,18], while biodistribution studies demonstrate that disease state and surface functionality alter localization [19,20,79]. The useful comparison is therefore not simply polymer versus lipid versus inorganic carrier but which attribute controls the rate-limiting loss of effective exposure.

Representative studies provide quantitative anchors with explicit boundary conditions. Avidin, a cationic protein carrier of approximately 7 nm with an isoelectric point (pI) near 10.5, showed an equilibrium dissociation constant of approximately 150 μM for cartilage, about 400-fold greater uptake than a similarly sized neutral control, and prolonged explant retention [23]. In a multi-arm avidin system, equilibrium cartilage uptake was approximately 112-fold the bath concentration in healthy cartilage and 33-fold in 50% glycosaminoglycan-depleted cartilage; a mixed linker design produced 50% dexamethasone release at 38.5 h and sustained release for approximately two weeks [24]. These values are useful starting points for charge-mediated carriers, but they remain specific to carrier architecture, payload, matrix state, and assay conditions.

The same logic extends beyond any single chemistry or payload. Self-assembling particles can retain anti-inflammatory proteins [80], depot-forming nanoparticles can prolong local exposure [81], and kartogenin-conjugated chitosan particles can couple localization to a defined chondrogenic cue [82]. These examples should be compared by exposure gained per unit of added complexity, including the amount of active payload delivered to cartilage rather than the total dose remaining anywhere in the joint.

The main design risk is to solve one transport barrier by spending the available binding and safety budget. A highly adhesive particle may remain in the joint yet fail to reach deep cartilage; a small penetrative carrier may distribute efficiently but clear rapidly; and a strongly cationic surface may improve matrix interaction while increasing synovial uptake or cytotoxicity. Intra-articular targeting studies therefore favor reversible interaction rather than maximal binding [83,84,85]. This trade-off should be mapped as a design window, not hidden behind a single optimized formulation.

A mechanistic transport analysis should move beyond nominal particle diameter and zeta potential. At minimum, studies should report size distribution in formulation and synovial-fluid-like media, free and protein-bound fractions, cartilage partition coefficient, depth-dependent concentration, effective diffusivity (D_eff), apparent association and dissociation, intact-carrier persistence, and bioactive cargo over time. An equilibrium dissociation constant (K_D) must not be relabeled as a dissociation rate: the association and dissociation rate constants (k_on and k_off) should be estimated separately where possible under relevant ionic strength, protein content, and matrix conditions. As an initial screening heuristic, the dissociation timescale (τ_off = 1/k_off) can be compared with the diffusion timescale (τ_diff ≈ L^2^/D_eff), while also accounting for k_on and available binding-site density. Very short τ_off relative to transport and dosing times risks inadequate retention, whereas very long τ_off with rapid association and high site capacity favors superficial sequestration. A candidate window is defined by target-depth exposure and reversible redistribution, not by a transferable k_off range. Measurements should include intact and matrix-depleted cartilage when disease-related glycosaminoglycan loss is relevant. Figure 3 illustrates the distinction between reversible penetration and surface trapping.

Stimuli-responsive carriers add temporal coupling between the material and its pathological environment. Reactive-oxygen-species (ROS)-, pH-, or enzyme-responsive systems can localize release to inflammatory conditions, but responsiveness should not be inferred from trigger exposure in buffer alone. Activation threshold, response kinetics, reversibility, payload stability, and off-target activation in synovium or subchondral bone must be measured. Cartilage-targeting ROS-responsive microspheres illustrate this opportunity [46], but disease-relevant validation is required before responsiveness can be interpreted as biological specificity.

### 4.2. Nano-Enabled Hydrogels and Mechanopharmaceutics

Hydrogels are not only local depots; they define the transport and mechanical boundary conditions experienced by nanocarriers, cargo, and cells. In situ gelation can accommodate irregular defects, whereas network density, mesh size, ligand presentation, and degradation control diffusion, cell invasion, and matrix deposition [36,65,86,87]. A hydrogel should be described as nano-enabled only when a nanoscale constituent or network feature produces a demonstrable change in release, rheology, toughness, cell signaling, imaging, or tissue interaction. Static modulus alone is an incomplete formulation descriptor.

Stress relaxation is particularly important because it can be varied independently of initial elastic modulus and can regulate cell spreading, proliferation, matrix deposition, and lineage decisions through adhesion-ligand clustering and actomyosin remodeling [88,89]. As one cartilage-oriented quantitative anchor, an injectable biphasic hyaluronic-acid system reported half-stress-relaxation times of approximately 60–2000 s at initial moduli near 3 kPa, with composition-dependent chondrogenic responses [90]. This span should not be generalized as an acceptance range. Faster relaxation is not intrinsically beneficial: a highly permissive matrix may lose shape or load support, whereas a slowly relaxing network may preserve geometry but constrain cell expansion and pericellular matrix assembly. The suitable timescale must be defined against cell source, repair phase, regional function, degradation, and cyclic-load stability.

Dynamic loading further couples matrix mechanics to mechanosensitive ion channels. TRPV4 and PIEZO1 can produce distinct chondrocyte transcriptional programs [67,68], so hydrogel evaluation should distinguish equilibrium modulus, dynamic modulus, relaxation time, permeability, and applied loading waveform. Reporting only a single compressive modulus obscures whether cells experience sustained confinement, transient fluid pressurization, or injurious strain concentration.

Nanocomposite hydrogels can modify these properties through fillers that alter rheology, toughness, mineral signaling, oxidative regulation, or imaging contrast. Clay and other nanofillers demonstrate how mechanics and bioactivity can be coupled [91,92], while reinforced and hyaluronic-acid-based matrices show how architecture can be adapted to cartilage and osteochondral defects [93,94,95]. Poly(ethylene glycol)–silicate and nanosilicate systems further illustrate growth-factor-free osteogenic instruction [96,97]. These advantages must be balanced against filler aggregation, local stress concentrations, altered degradation, and inappropriate mineral signaling in cartilage-facing regions.

Mechanical and pharmaceutical functions should be evaluated under coupled conditions. In a rare direct example, a mechanically resilient TG-18 hydrogel was tested by rotational rheometry under alternating low-load conditions (0.5% strain, 0.1 Hz) and conditions selected to simulate running (35% strain, 2.5 Hz) for 30 one-minute high/low cycles; it recovered viscoelastic structure and maintained release, whereas a comparator gel failed to recover and showed loading-altered release [49]. Matched running and non-running mouse experiments then linked loading-resilient delivery to local drug exposure and therapeutic response. This study provides an operative test pattern, but its shear waveform, single formulation, and single payload do not establish a transferable cartilage-compression protocol. Future studies should compare static and loaded release under fully reported compression or shear amplitude, frequency, duty cycle, contact geometry, temperature, and synovial-fluid-like medium, while co-measuring storage and loss moduli (G′ and G″) or dynamic modulus, deformation and recovery, mass loss, carrier integrity, and bioactive payload.

Tribological function is an additional design requirement for materials exposed at the articular surface. Low friction depends on hydration, fluid pressurization, and boundary-lubricating molecules rather than stiffness alone. Cartilage-like coatings and polyelectrolyte-based hydration layers can reduce friction under aqueous loading [98,99], while hydration-lubrication and lubricin studies show that lubricant dose, surface retention, and rehydration behavior influence the mode of lubrication [100,101]. Candidate surface materials should therefore be tested for friction, wear, rehydration, and debris generation against opposing cartilage under physiologically relevant contact pressure and sliding cycles.

The broader hydrogel literature supports a minimum-complexity principle. Crosslink chemistry, ligand presentation, nanofiller content, and degradation should each be retained only when they regulate a prespecified interaction [87,102,103]. Cartilage-focused studies emphasize mechanical compatibility and phenotype stability [104,105,106], whereas injectable and biodegradable systems highlight handling and manufacturability [107,108]. A multifunctional hydrogel is valuable only when every module resolves a measurable barrier without impairing transport, remodeling, sterility, or batch control.

### 4.3. Defining Context-Specific Design Windows

Published values should be treated as quantitative anchors rather than generic specifications. There is no evidence-based particle-size interval, surface-charge range, affinity constant, or stress-relaxation time that is optimal across a soluble drug, extracellular vesicle, cartilage-penetrating carrier, and defect-filling depot. Each candidate range requires two-sided failure boundaries: insufficient interaction or persistence at one extreme, and aggregation, surface trapping, synovial sequestration, cytotoxicity, impaired remodeling, or mechanical failure at the other.

A practical workflow begins by specifying the target compartment, depth, exposure duration, payload activity, lesion matrix state, and loading regime. Factorial or response-surface screens should then vary a small set of material attributes while measuring particle distribution, cartilage partitioning and depth, D_eff, k_on/k_off where applicable, synovial uptake, active-cargo exposure, cytotoxicity, release, and material recovery. The non-dominated region that satisfies the exposure target without crossing a prespecified safety or mechanical failure boundary becomes the provisional design window. That window should be challenged across healthy and depleted matrices, disease-relevant fluids, loading, sterilization, storage, and representative batches before it is converted into a critical quality attribute (CQA) acceptance range.

Every reported window should state formulation state, analytical method, medium, temperature, tissue source and thickness, dose, loading waveform, and uncertainty. A single optimized formulation is not a window; at least one failing condition on each side is needed to establish causality. Table 3 summarizes published anchors and the decisions they can inform while making their limits explicit.

## 5. Nano–Bio Interfaces, Cell-State Control, and Bioinspired Nanobiologics

### 5.1. Protein Corona, Host Response, and Cell-State Control

Biological evaluation of regenerative nanomaterials should follow a claim hierarchy: compatibility, local pharmacology, cell-state correction, tissue formation, and, finally, functional regeneration. Viability and proliferation support only the first level. Single-cell studies show heterogeneous repair, inflammatory, fibrotic, and hypertrophic states [62,63], while extracellular-vesicle studies show that complex cargo can redirect repair-relevant signaling [110,111,112]. The central question is whether material and cargo jointly move resident or recruited cells toward stable repair states without fibrosis, hypertrophy, persistent inflammation, or ectopic mineralization.

The biomaterial–host interface is shaped by protein adsorption, surface chemistry, particle stiffness, charge, ligand density, topography, degradation products, and inflammatory context. In synovial fluid, a disease-dependent protein corona can mask targeting ligands, change apparent surface charge, and redirect uptake; direct cartilage-uptake studies confirm that the synovial-fluid profile can determine nanoparticle behavior [113]. Material evaluation should therefore measure corona formation, synovial uptake, cartilage penetration, and immune-cell interaction in disease-relevant fluids rather than infer them from serum-free media.

Material-driven host responses should be resolved from cargo-driven pharmacology. Blank carriers, unloaded hydrogels, and degradation products require their own controls, and immune readouts should include synovial barrier integrity, macrophage-state trajectories, fibroblast-like synoviocyte activation, and foreign-body-cell accumulation. Systems that improve cartilage histology while producing persistent synovial inflammation or wear-particle uptake have not achieved a compatible host response. Immunomodulatory hydrogels and microspheres provide useful proof of principle, but their mechanism should be tested with temporal cell-state analysis rather than endpoint cytokine panels alone [57,58,59,60,61].

Cell-state-oriented endpoints should integrate anabolic, catabolic, hypertrophic, and spatial markers with matrix composition and mechanics. A material that increases COL2A1 while also inducing COL10A1 and RUNX2 may not produce stable cartilage. Likewise, reduced inflammatory cytokines do not establish immune resolution if synovial barrier function, macrophage-state transitions, and matrix homeostasis remain abnormal [55,56,62,63,64].

### 5.2. Extracellular Vesicles as Nanobiologics: When Biological Complexity Is Justified

Extracellular vesicles (EVs) provide a distinct bioinspired biomaterial strategy. Unlike synthetic carriers that transport a defined payload, EVs contain combinations of microRNAs, proteins, lipids, and metabolites that can regulate recipient cell states. The 2018 and 2023 Minimal Information for Studies of Extracellular Vesicles (MISEV) guidelines define the minimum evidence required to identify and characterize EV preparations [37,114], while broader biological reviews describe their physiological functions and cell-interaction mechanisms [115,116,117]. Vonk et al. provided early evidence that mesenchymal stromal cell-derived EVs can promote human cartilage regeneration in vitro [110].

Subsequent work has linked EV composition and donor state to repair mechanisms. Chondrogenically primed EVs enriched in miR-455 activate the SRY-box transcription factor 11/forkhead box O (SOX11/FOXO) axis [111], bioenergetically active exosomes support cartilage regeneration and homeostasis [118], and adhesive hydrogels can localize EVs within articular defects [112]. More recent preclinical evidence shows that small EVs from induced-pluripotent-stem-cell-derived chondrocytes can promote repair-relevant responses in osteoarthritic cartilage models [119]. These studies indicate that EV efficacy depends on both biological cargo and the material environment that controls retention, presentation, and recipient-cell access; they do not by themselves establish manufacturing readiness or comparative clinical superiority.

The same complexity that gives EVs broad biological activity also creates uncertainty. Donor cell type, preconditioning, culture conditions, isolation method, particle-size distribution, cargo composition, storage, and potency assays can alter function [37,114]. Platelet-derived vesicles illustrate how an alternative donor source changes both the biological premise and the required identity controls [120]. EV-loaded bioinks and decellularized-matrix constructs illustrate spatial delivery opportunities [121,122,123], whereas mechanism and disease-model studies show the need to define active components and response pathways [124,125,126]. EVs should therefore be treated as engineered nanobiologics with measurable identity and potency, not as intrinsically superior natural particles.

Barrier-guided selection requires an operational mechanism-necessity test. Coordinated signaling is not established by detecting two or more cargo species. At matched target-cell exposure, intact EVs should be compared with a disrupted or cargo-depleted EV preparation, the leading single active cargo, and a defined synthetic cargo combination. Candidate components should be perturbed by depletion, inhibition, or recipient-pathway blockade, and the response should be tested for restoration or interaction beyond the single-cargo effects. A batch-level potency assay should then correlate with a downstream repair-relevant output, such as stable cartilage matrix production without hypertrophy, across independently manufactured lots. Whole-EV complexity is justified only when it reproducibly outperforms the simpler comparator, the responsible activity survives incorporation and release from the delivery matrix, and the potency specification predicts the claimed function [37,110,111,112,114,115,116,117,118,119].

### 5.3. Function-Matched Selection of Synthetic and Natural Carriers

Poly(lactide-co-glycolide) (PLGA) and hyaluronic acid (HA) solve different formulation problems and should not be ranked as interchangeable carrier classes. PLGA is favored when encapsulation of a defined payload, particle processability, and hydrolysis-controlled release are dominant requirements, but acidic degradation products, burst release, and phagocytic sequestration must be controlled. HA is favored when matrix-mimetic interaction, cluster of differentiation 44 (CD44)-associated cell engagement, hydration lubrication, or an injectable and remodelable network is central, but enzymatic degradation, cargo escape, and molecular-weight-dependent biology must be qualified. A PLGA–HA hybrid is rational only when separating core release control from surface/tissue interaction produces an exposure advantage over both single-component controls. Direct comparison of PLGA with HA-grafted PLGA, for example, showed increased chondrocyte and cartilage association after HA functionalization, but it did not establish general superiority of HA or PLGA [127].

A valid side-by-side comparison should hold payload, active dose, particle geometry or depot volume, administration method, medium, disease state, and observation time constant. It should report recovered dose, release, intact-carrier fate, cartilage depth distribution, synovial uptake, target-cell exposure, potency, degradation products, and manufacturing variability. If these variables are not matched, material-class comparisons should be described as contextual examples rather than performance rankings.

Figure 4 provides a qualitative evidence map across adhesive materials, mechano-adaptive hydrogels, and gradient or layered constructs. Symbols represent the apparent directness and convergence of evidence within each strategy–validation-domain pair; they do not encode pooled effect magnitude, formal certainty, or comparative superiority. Table 4 summarizes the principal functions and failure risks of major nano-enabled modules, and Table 5 links representative studies to structure–property–exposure insights and unresolved questions.

## 6. From Nanoscale Delivery to Multiscale Osteochondral Reconstruction

### 6.1. Zone-Specific Biology and Nano-Enabled Cue Control

Osteochondral regeneration requires restoration of spatial tissue identity. The cartilage zone should remain avascular and non-mineralized while producing a proteoglycan-rich type II collagen matrix; calcified cartilage must provide a controlled mineral–organic transition; and subchondral bone requires vascularized mineralized tissue with sufficient mechanical support [5,69,75]. These regions are not independent layers but a mechanically coupled interface.

Single-phase materials rarely satisfy these competing requirements. Gradient and multilayered systems establish regional architecture [40,69,70,71,72], while bioprinting and woven constructs provide routes to mechanically integrated repair [39,73,74,75]. The relevance to nanomaterial design is multiscale: nanoscale carriers and topographies regulate local cue exposure and cell interaction, whereas the macroscale construct preserves defect geometry and load transfer. A uniformly chondrogenic matrix may fail on the bone-facing side, while a highly mineralized scaffold may promote cartilage hypertrophy. Spatial repair therefore requires cue confinement, synchronized degradation, and continuous load transfer rather than visual layering alone [76].

Cargo and carrier selection should follow spatial biology. Cartilage-facing regions may benefit from hyaluronic-acid matrices, chondrogenic peptides, penetrating nanocarriers, or EV-based signals [66,95,111], whereas bone-facing regions may require mineral or osteogenic cues supplied by clay, nanosilicate, or related matrices [69,92,97]. The interface should provide a gradual transition in mineralization, stiffness, degradation, and release rather than an abrupt boundary, as illustrated by woven and human-scale printed constructs [74,75].

### 6.2. Architecture, Interface Mechanics, and Validation

Bilayer, trilayer, gradient, and three-dimensional printed constructs provide manufacturing routes for spatial repair, but only some are functionally nano-enabled. Nanoscale topography, nanofiller gradients, or localized nanocarriers should be retained when they alter cell adhesion, cue confinement, mineral transition, or interface mechanics [40,69,70,71,72]. Woven architectures and bioprinting can establish tissue-scale geometry [39,73,74,75]; the central question is whether nano- and macroscale features remain coupled during implantation and remodeling.

Regional stiffness and cell organization can be engineered through gradients and printing [71,73]. Spheroid microchambers, hybrid constructs, progenitor-cell bioinks, and cell electrowriting provide additional control over cellular organization [128,129,130,131], while developmentally inspired templates and tissue-derived matrices extend spatial design toward tissue-specific maturation [132,133]. A multilayered construct is not functionally spatial unless local cues remain within the intended region and the interface resists delamination under load.

Interface integration depends on more than a gradual composition profile. Interdigitating pores, fiber continuity, interfacial crosslinks, controlled mineral transitions, and regional topography can redistribute shear and reduce delamination, whereas abrupt modulus changes concentrate stress at the cartilage–bone boundary [40,134]. Anatomical scaffold design should therefore specify how adjacent phases are joined and how this junction evolves during degradation. The relevant failure tests include dry and hydrated interface strength, push-out or lap-shear testing, cyclic compression, fatigue crack initiation, and post-degradation mechanical continuity.

Validation should therefore be quantitative, depth-resolved, and mechanical. Minimum readouts include cargo concentration as a function of depth and time; cross-layer leakage; cartilage-zone COL2A1, ACAN, and proteoglycan deposition; interface COL10A1 and mineral-gradient continuity; bone-zone RUNX2, osteocalcin, and micro-computed-tomography bone volume; and interfacial push-out or shear strength. Cyclic compression, fatigue, and delamination testing should accompany spatial molecular mapping [69,71,73,74,75].

Large-animal studies are particularly informative because clinically relevant defect diameter, cartilage thickness, and joint loading expose design failures that may remain hidden in rodents or rabbits. Recent sheep studies show that injectable or printed biomaterials can be evaluated under demanding articular loading, while multilayered constructs reveal the need to couple regional tissue formation with interface durability and surgical handling [135,136,137]. These models should be used to test a specific barrier claim rather than merely repeat small-animal histology at a larger scale.

The spatial design logic for osteochondral interface reconstruction is summarized in Figure 5. Biomimetic architecture and three-dimensional bioprinting are presented as complementary routes to combine anisotropic mechanics, regional cell and matrix organization, and restoration of cartilage, calcified cartilage, and subchondral bone.

## 7. In Vivo Fate, Local Pharmacokinetics, and Mechanistic Validation

The in vivo fate of regenerative nanomaterials is often insufficiently connected to active exposure and efficacy. Nanoparticles may remain in the defect, migrate to synovium, penetrate cartilage, enter subchondral bone, or redistribute systemically. Hydrogels may preserve gross shape while losing cargo, and scaffolds may degrade before replacement tissue supports load. Imaging and tracking can resolve localization [138,139,140], but mechanistic interpretation requires concurrent measurement of intact carrier, released payload, material transformation, and biological response [141,142,143].

Imaging-enabled materials can help address this uncertainty. Iron oxide nanoparticles can support magnetic-resonance tracking, fluorescent particles can map distribution in small-animal models, and photoacoustic or multimodal probes can report tissue localization [138,139]. Such signals are useful only when the label does not materially change dispersion, mechanics, degradation, or cell interaction.

Imaging signal should not be equated with material integrity, payload activity, or regeneration. A label can persist after cargo release or carrier degradation, and a retained scaffold can remain biologically ineffective. Local pharmacokinetic/pharmacodynamic analysis should therefore connect cavity concentration, cartilage depth distribution, cellular exposure, cargo activity, matrix response, mechanical integration, and safety [138,139,140,141,142,143].

Fluorescence, magnetic resonance imaging (MRI), and positron emission tomography (PET) answer different fate questions and fail in different ways. Fluorescence is sensitive and practical for serial small-animal imaging but is affected by tissue attenuation, photobleaching, spectral overlap, and label transfer. MRI provides deep anatomical context without ionizing radiation, yet has lower molar sensitivity; iron-based labels may persist after carrier degradation or transfer to phagocytes, and negative contrast can be nonspecific. PET enables sensitive, quantitative whole-body measurement, but spatial resolution is limited and radionuclide detachment, metabolite redistribution, and physical decay can uncouple signal from carrier integrity. For each modality, label–carrier linkage and labeling-induced changes in size, mechanics, release, and potency should be established, and imaging should be paired with an orthogonal assay for intact carrier and active payload in the target tissue [49,138,139,140].

Serial analysis should distinguish material transformation from disappearance. Swelling, protein adsorption, oxidation, hydrolysis, mineral exchange, and mechanical fragmentation can change the delivered material state before mass is cleared. Retrieval studies and time-resolved chemical, mechanical, and histological analyses are needed to determine whether loss of signal reflects degradation, redistribution, phagocytic uptake, or tissue replacement.

Cross-species dose scaling should report at least injected mass per joint, mass per synovial-fluid volume, mass per cartilage surface area or defect volume, and the resulting cartilage concentration–time profile. Body-surface-area allometry may inform systemic safety, but it is not sufficient for intra-articular exposure. Cartilage transport time depends strongly on diffusion distance: in one avidin model, medial tibial cartilage half-life was approximately 154 h in rabbits versus 29 h in rats, and normalization by cartilage thickness squared increased the correlation with retention from R^2^ = 0.43 to 0.96 [109]. This is a carrier-specific anchor, but it shows why translation should target comparable tissue area under the concentration–time curve (AUC), depth distribution, and exposure duration rather than an identical dose per kilogram.

## 8. Translational Pharmaceutics: Critical Quality Attributes, Safety, and Model Relevance

### 8.1. Critical Material Attributes and Functional Identity

For local nanopharmaceutic systems, identity includes more than nominal chemical composition. Critical quality attributes (CQAs) should be selected because they govern effective exposure or repair function. Nanocarriers therefore require particle-size distribution, zeta potential, morphology, surface chemistry, ligand accessibility, cargo loading, dose recovery, release, and stability in synovial-fluid-like conditions. A mean diameter can conceal a broad, skewed, multimodal, or aggregate-containing distribution. The polydispersity index (PDI) is a cumulants-based, batch-sensitive summary for approximately unimodal dynamic light scattering (DLS) data; it does not represent an independent biological mechanism and should not replace the distribution itself. For broad or multimodal populations, method-qualified D10, D50, and D90 values or modal distributions together with orthogonal particle imaging or separation should be reported. PDI becomes a CQA only when its change predicts aggregation, cartilage partitioning, synovial uptake, safety, release, or potency [22]. Hydrogels and scaffolds likewise require network architecture, viscoelasticity, fatigue, degradation, filler dispersion, and spatial integrity [22,78].

Material attributes are mechanistic variables rather than supplementary descriptors. Acceptance ranges should be derived from two-sided functional boundaries, not borrowed from generic examples such as 50–500 nm or 80–150 nm. Particle size, charge, and affinity ranges should be challenged until loss of cartilage access, aggregation, excessive synovial uptake, surface trapping, or cytotoxicity is observed [17,18,23,24]. Hydrogel ranges should similarly be bracketed by failure of injection or retention, release, cell remodeling, fatigue, or load support [36,47,86,87,88,89,90]. Candidate specifications should then incorporate analytical uncertainty, interbatch variability, storage and sterilization drift, and process capability while remaining inside the exposure and potency window.

Acceptance criteria should reflect the intended clinical scenario. Injectable systems require reproducible syringeability, delivered-dose recovery, and gelation under operative conditions; exposed cartilage materials require friction and wear testing; load-bearing constructs require dynamic mechanics, interface strength, and fatigue; and delivery systems require stability and release in synovial-fluid-like media under matched static and cyclic loading. A proposed range is credible only when excursions on both sides predict a prespecified loss of exposure, safety, or function and when qualified methods can distinguish that loss. Quality-by-design should preserve the causal structure–property–exposure–function relationship [21,22,78].

During development, the experimentally supported operating region should not be adopted directly as a routine release specification. After analytical-method qualification, the acceptance interval should remain inside both functional failure boundaries, include a predefined guard band for measurement uncertainty and storage or process drift, and be supported by between-batch tolerance intervals and process-capability analysis. Until these product-specific data exist, published values can justify screening levels but not numerical lot-release limits [22,78]. Table 6 summarizes the mechanism-linked CQAs and the basis for product-specific acceptance ranges.

### 8.2. Dose, Sterilization, Storage, Degradation, and Long-Term Safety

Scale-up, sterilization, storage, and administration can alter particle size, ligand presentation, EV potency, network mechanics, nanofiller dispersion, and delivered dose. Translation analyses identify these manufacturing gaps [21,77], while clinical nanomedicine reviews show how incomplete fate and safety characterization delays development [141,142,143]. Post-process testing should therefore repeat the CQAs linked to mechanism, quantify dose recovery through the intended delivery device, and verify that the exposure window is preserved.

Long-term safety depends on degradation, persistence, and host response. Short-term histology cannot exclude delayed inflammation, synovial fibrosis, immune activation, wear-particle generation, or off-target accumulation, particularly for persistent inorganic or mineralized components [21,22,78,141,142,143]. Degradation products should be connected to local pH, inflammatory signaling, mechanical loss, and systemic distribution.

### 8.3. Minimum Necessary Complexity and Development-Stage Model Relevance

Increasing material complexity can obscure causal attribution and create additional failure modes. A platform that combines targeting ligands, responsive chemistry, nanoparticles, EVs, and a multiphasic scaffold may be biologically attractive but difficult to dose, characterize, sterilize, and reproduce. We therefore propose a complexity budget: every added module should resolve a prespecified barrier, provide incremental benefit over a simpler comparator, and retain a measurable CQA linked to function.

The active cue and the material platform should be evaluated separately before their interactions are optimized. When a defined small molecule or protein is biologically sufficient, a well-characterized carrier or scaffold can simplify attribution of potency, toxicity, and degradation. More complex nanobiologics are justified only when coordinated cargo or membrane-mediated interactions provide a necessary biological function that simpler systems cannot reproduce [22,78].

Regulatory identity should be considered while the platform is still being designed. Depending on primary mode of action and claims, a scaffold without active cargo may follow a device-oriented pathway, a nanocarrier containing a defined drug may be developed as a medicinal product, and a drug- or biologic-loaded scaffold may be a combination product [22,144]. An EV-in-hydrogel hybrid requires integrated controls across both constituents: donor and culture history, isolation, and EV identity before incorporation; EV recovery, integrity, cargo accessibility, and potency after release; hydrogel gelation, mechanics, degradation, and EV–matrix interaction; and aseptic processing, storage, container closure, and delivered-dose recovery. A potency assay should be capable of detecting batch-to-batch loss caused by either EV manufacture or the matrix. Because classification and lead-center assignment are jurisdiction- and product-specific, early regulatory interaction should test the proposed primary mode of action and define which constituent-part and combination-specific studies are required [37,114,144].

The minimum-complexity principle does not imply that simple materials are always superior. Cartilage-targeted carriers can solve tissue access [17,18], dynamic hydrogels can coordinate mechanics and local release [36,86], EV-based systems can reprogram complex cell states [111,112], and spatial constructs can reconstruct zonal interfaces [74,75]. The appropriate level of complexity is the lowest one that preserves the required mechanism across manufacture, administration, loading, degradation, and remodeling.

Preclinical model selection must reproduce the barrier being claimed. Young, healthy small-animal defects do not capture human cartilage thickness, chronic inflammation, joint loading, or comorbid degeneration [5,145]. Large-animal studies are not merely a later efficacy step; they test whether delivery depth, material fatigue, interface stability, synovial safety, and surgical handling remain coupled at a clinically relevant scale. Sheep studies of supramolecular/covalent scaffolds, transforming growth factor beta (TGF-β)-functionalized printed constructs, and multilayered osteochondral scaffolds illustrate how material retention, regional repair, and load-bearing performance can be interrogated in larger defects [135,136,137]. Model selection should be justified by the transport, host-response, or mechanical barrier under study, with follow-up long enough to evaluate degradation and tissue replacement. Figure 6 integrates these requirements into a quality-controlled translational pathway from preclinical limitations and critical material attributes to large-animal validation, clinical readiness, regulatory clarity, and scalable manufacturing.

## 9. Critical Synthesis: Design Rules, Trade-Offs, and Unresolved Questions

The central argument of this review is that nano-enabled cartilage repair should be judged by a causal chain: critical material attribute, delivered material state, effective tissue exposure, cell-state response, and functional repair. Cartilage-targeted particles show how surface properties govern access [17,18]; hydrogels define mechanical and transport microenvironments [36,86]; EVs couple complex cargo to cell-state regulation [110,111,112]; and spatial constructs address conflicting cartilage and bone requirements [74,75]. The framework connects these platforms without assuming that any class is intrinsically superior.

The first design rule is that nano-enablement must be causal. A nanoscale filler, surface feature, or carrier should be retained only when it changes a limiting transport, release, mechanical, or cell-interaction process relative to a suitable comparator. Particle size or nanostructure is a material description; it becomes a therapeutic design variable only when the resulting function is demonstrated [17,18,23,24].

The second design rule is that effective tissue exposure precedes biological potency. Total dose remaining in the joint is not an adequate surrogate for active cargo in cartilage. Cavity persistence, tissue partitioning, penetration, release, and cellular bioavailability should be resolved separately, and reversible binding should replace maximal affinity as the default objective [17,18,23,24].

The third design rule is that mechanics are part of the formulation. Loading alters depot shape, fluid exchange, release, friction, and cell mechanotransduction. Stiffness, viscoelasticity, permeability, degradation, and loading waveform should therefore be interpreted jointly rather than as independent material and biological variables [55,62,65,86,87,88,89,90].

The fourth design rule is that regeneration claims require cell-state and functional evidence. Increased COL2A1 or glycosaminoglycan content is encouraging, but stable repair also requires exclusion of fibrotic and hypertrophic trajectories, restoration of depth-dependent matrix organization, appropriate mechanics, and integration. Reduced inflammation alone should be described as disease-modifying pharmacology unless structural regeneration is demonstrated.

The fifth design rule is that spatial architecture must be validated as interface function. Multilayered appearance is insufficient when signals leak across zones, degradation is asynchronous, or the cartilage–bone interface delaminates under load. Spatial molecular mapping should be interpreted together with dynamic mechanics, interface strength, and long-term tissue replacement [69,70,71,73,74,75].

The sixth design rule is that material identity and complexity must remain controllable. Sterilization, storage, protein adsorption, degradation, and mechanical loading can transform a platform after initial characterization [21,22,77,78]. Numerical targets should therefore be expressed as context-dependent design windows with two-sided failure boundaries, and every added module should earn its complexity through incremental function and a corresponding CQA. Table 7 summarizes the measurements required to test these rules.

### Limitations and Evidence Gaps

The framework has three important limitations. First, the quantitative values assembled here are platform-specific anchors, not validated universal specifications; clearance, penetration, inflammation, mechanics, and spatial mismatch vary with lesion geometry, matrix depletion, loading, payload, and time. Second, direct head-to-head comparisons of synthetic and natural carriers are uncommon and often confounded by different payloads, doses, geometries, or endpoints. Third, only a small body of mechanopharmaceutical literature simultaneously measures loading waveform, material-state recovery, and active-cargo release under cartilage-relevant conditions. Most studies also omit one or more steps of the local pharmacokinetic cascade, so cavity retention is still overinterpreted as target exposure. Barrier prioritization and the proposed design-window workflow therefore remain testable development hypotheses rather than standardized algorithms.

These gaps define a focused research agenda. Prospective factorial studies should vary size distribution, charge and affinity kinetics, stress relaxation, degradation, or interface architecture while holding payload and administration conditions as constant as practicable. Loading studies should predefine static and physiological-waveform comparators and co-measure mechanics, deformation, intact carrier, active payload, and biological response. Carrier comparisons should use exposure-matched PLGA, HA, hybrid, or EV systems and include simpler mechanism controls. Finally, multicohort and multibatch studies should deliberately sample both sides of a provisional window so that CQA acceptance limits can be estimated from exposure, potency, safety, and process capability rather than from a single optimized formulation.

## 10. Future Perspectives: Toward Predictive and Stage-Adaptive Nanomaterial Design

Future progress should come less from adding functions and more from quantifying how material attributes change delivered state, exposure, and repair trajectories. Six priorities emerge.

First, studies should begin with a barrier and exposure profile rather than a preferred material. Joint-clearance problems require separate measurements of cavity residence and synovial uptake; cartilage-penetration problems require depth-resolved size, charge, deformability, and affinity analyses [17,18,84,85]; and unstable defects require dynamic localization and fatigue testing before biological optimization.

Second, nanomaterials should be matched to repair stage. Early formulations may require rapid inflammatory modulation and permissive remodeling, whereas later phases require sustained matrix assembly, increasing mechanical competence, and spatial integration. Responsive release, reversible binding, stress relaxation, and degradation should be evaluated as coordinated temporal variables [36,47,48,87,88,89,90]. Stage-adaptive design is valuable only when the trigger and transition are measurable rather than assumed [146].

Third, material design should target cell states rather than cell types alone. Single-cell and spatial approaches can identify inflammatory chondrocytes, hypertrophic progenitors, activated synovial cells, and abnormal bone-remodeling states. These states can guide ligand selection, cargo choice, mechanical cues, and potency assays [62,63,64].

Fourth, spatial biomaterials require direct validation of cue confinement and interface function. Depth-resolved release, regional molecular mapping, interfacial shear or push-out testing, and cyclic load-bearing assessment should become standard for multilayered, gradient, and topographically patterned constructs [40,69,70,71,72,73,74,75,76,134,135,136,137]. Large-animal studies should be designed around prespecified failure modes such as delamination, cross-layer leakage, insufficient cartilage thickness, or premature loss of load support.

Fifth, CQAs and clinically relevant mechanics should enter early development. Target product profiles should define acceptable ranges of particle state, surface chemistry, dose recovery, viscoelasticity, degradation, sterilization response, and storage stability that preserve effective exposure and biological function [21,22,77,78].

Sixth, high-throughput experimentation and interpretable machine learning can map multidimensional structure–property–exposure–function relationships. Libraries that vary particle size, charge, ligand density, network mechanics, degradation, and spatial architecture can be screened against barrier-specific readouts [147,148]. The immediate priority is not black-box optimization but prospectively designed experiments that reveal interactions, define failure boundaries, and generate reusable datasets.

## 11. Conclusions

Cartilage and osteochondral regeneration are constrained by coupled losses of exposure, compatible host response, mechanical function, and spatial organization. A barrier-guided approach connects each failure to a controllable nanomaterial attribute, the delivered state that emerges in the joint, an intended tissue interaction, a competing risk, and a decisive validation test.

Across material classes, the evidence supports context-dependent design windows rather than universal optima. Charge-mediated carriers provide useful quantitative anchors for reversible partitioning and penetration [17,18,23,24,44], but no transferable k_off or particle-size range has been validated. Nano-enabled hydrogels link relaxation and release to cell remodeling [36,67,68,86,87,88,89,90], whereas direct matched loading–release evidence remains limited [49]. EV nanobiologics require comparator-based evidence that multicomponent activity is necessary and a potency assay that survives matrix incorporation [37,110,111,112,113,114,115,119]. Spatial constructs must couple cue confinement to fatigue-resistant interface integration [39,40,69,70,71,72,73,74,75,76,134,135,136,137]. The next advance is therefore not a universal threshold table but reproducible methods that convert platform-specific anchors into failure-bounded specifications.

Progress will depend less on multifunctionality itself than on preserving a causal structure–property–exposure–function relationship through manufacture, administration, loading, degradation, and tissue replacement. The most credible nanomaterial is not the most elaborate one but rather the simplest platform that produces a necessary, measurable, and durable advantage at the limiting step of repair.

## Figures and Tables

**Figure 1:**
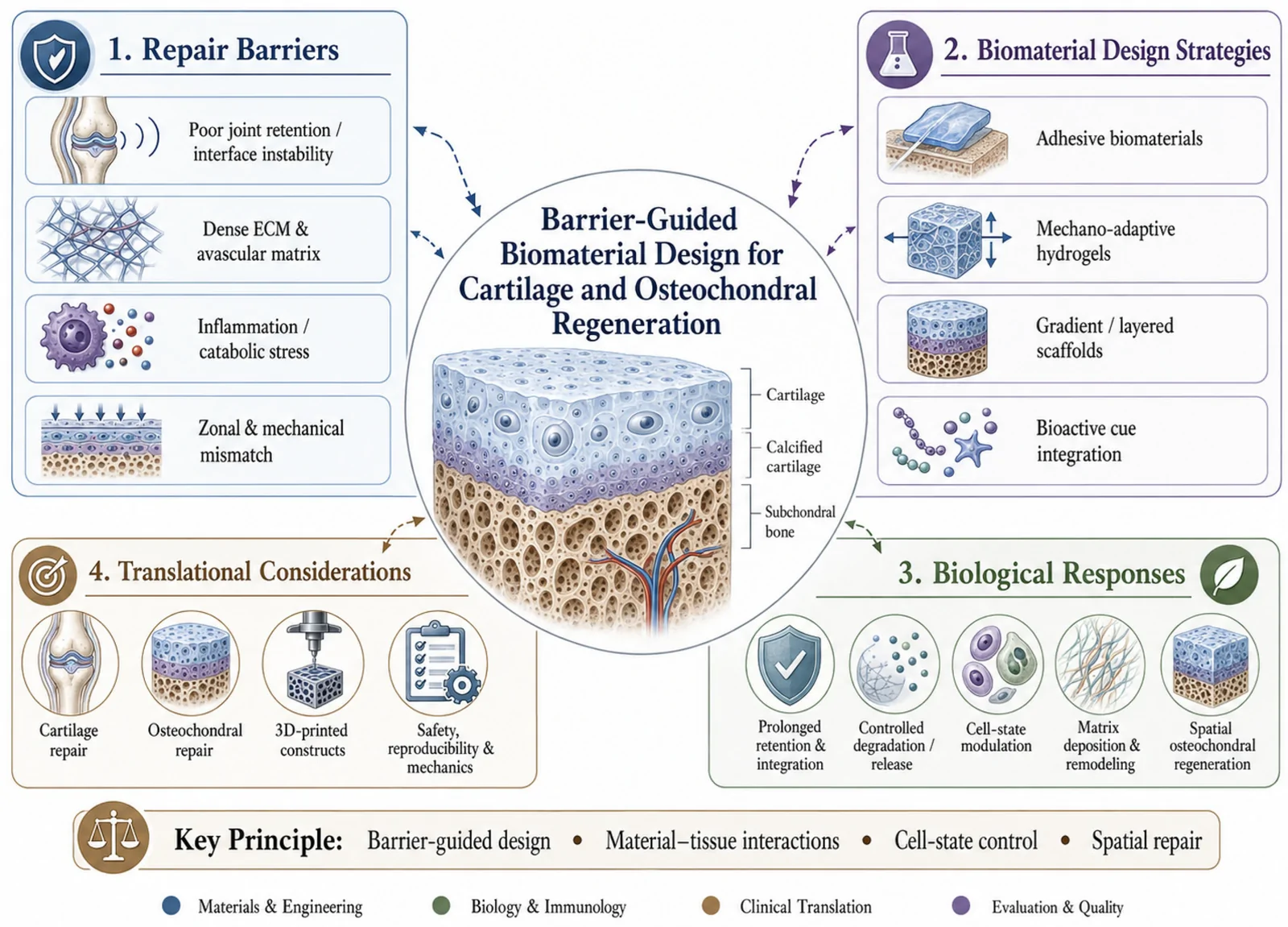
Barrier-guided nano-enabled biomaterial design for cartilage and osteochondral regeneration. Repair barriers include poor joint retention and interface instability, dense and avascular ECM, inflammatory and catabolic stress, and zonal or mechanical mismatch. Nano-enabled strategies should be judged by the causal sequence from material attribute and delivered state to effective exposure, cell-state response, spatial repair, and translational reproducibility. The framework organizes rather than ranks material classes [17,18,19,20,21,22,23,24,36,37,38,39,40]. Colored dashed arrows link the central framework to the four surrounding domains: blue, purple, green, and brown correspond to repair barriers, biomaterial design strategies, biological responses, and translational considerations, respectively.

**Figure 2:**
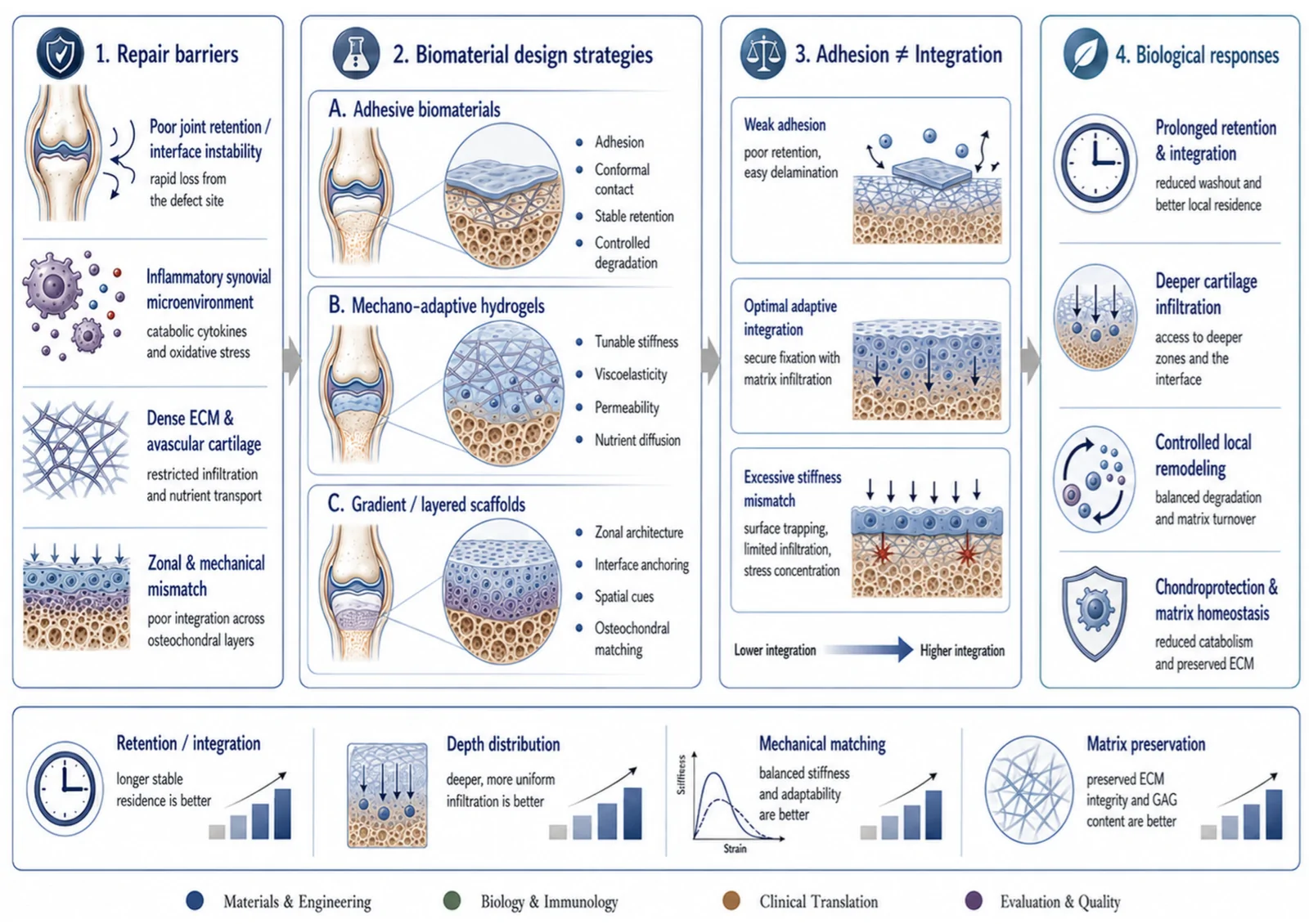
Integration-oriented nanomaterial design and mechanism-linked validation. Poor defect-site retention, inflammatory synovial conditions, dense avascular cartilage, and zonal or mechanical mismatch motivate adhesive materials, mechano-adaptive hydrogels, and gradient or layered constructs. The objective is adaptive integration rather than maximal adhesion or stiffness. Minimum validation domains include retention, cartilage depth distribution, release under loading, mechanical matching, and preservation of cartilage matrix [17,36,47,48,55,56,62,63,64,65,66,67,68,69,70,71,73,74,75]. This literature-informed conceptual schematic does not depict original experimental data. Grey arrows indicate the proposed progression from repair barriers through biomaterial design and adaptive integration to biological responses; in the integration panel, arrows distinguish weak adhesion, optimal adaptive integration, and excessive stiffness mismatch.

**Figure 3:**
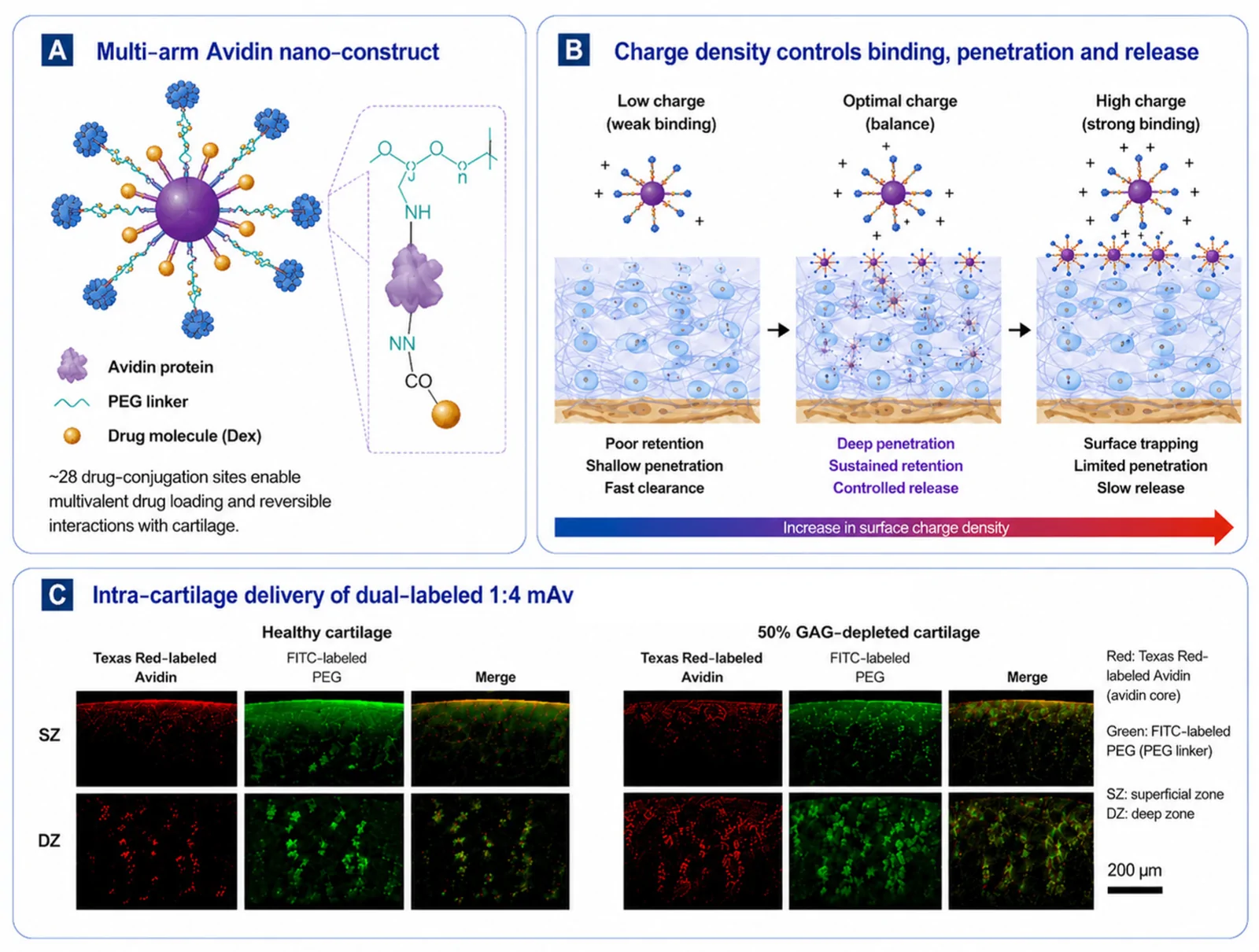
Charge-dependent intra-cartilage transport and retention of multi-arm avidin nano-constructs. (**A**) Schematic architecture of the multi-arm avidin nano-construct, comprising an avidin core, multi-arm poly(ethylene glycol) (PEG) linkers, and conjugated small-molecule drug molecules; dexamethasone (Dex) is shown as the representative payload. (**B**) Conceptual charge–penetration–retention trade-off. Insufficient positive charge results in weak cartilage binding and rapid clearance, whereas excessive charge or multivalent affinity can cause superficial sequestration. An intermediate reversible interaction supports full-depth penetration, sustained retention, and controlled release. (**C**) Representative confocal images of dual-labeled 1:4 multi-arm avidin (mAv) in healthy and 50% glycosaminoglycan (GAG)-depleted cartilage. Texas Red-labeled avidin is red, fluorescein isothiocyanate (FITC)-labeled PEG is green, and merged images show colocalization. SZ, superficial zone; DZ, deep zone. Panels (**A**,**B**) are author-prepared schematic interpretations based on [23,24,44,45]. Panel (**C**) is adapted from He et al. [24], (**C**). Scale bar, 200 μm. In panel (**A**), purple denotes avidin, turquoise denotes PEG linkers, and the yellow-orange spheres denote Dex.

**Figure 4:**
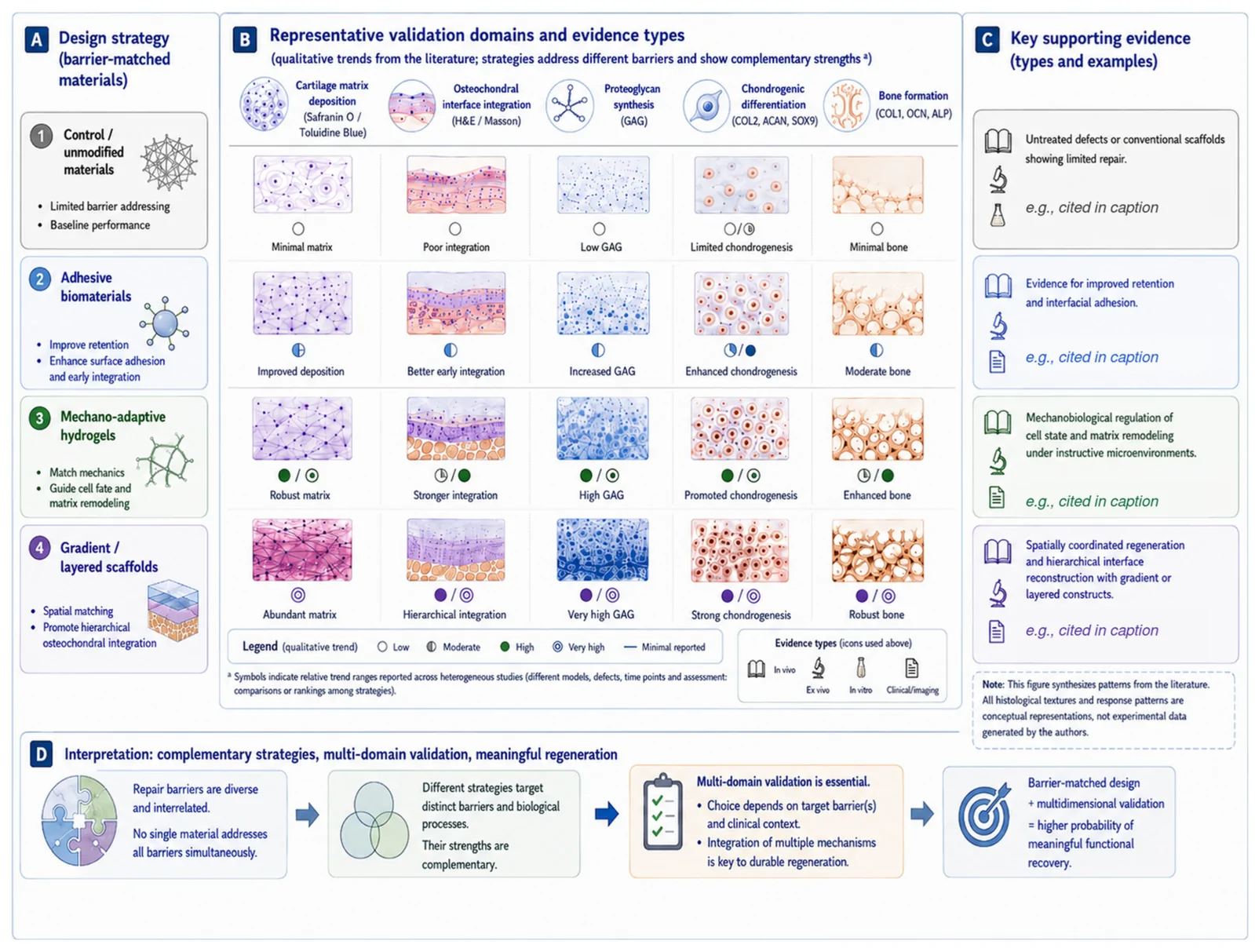
Literature-informed synthesis of complementary material strategies across validation domains. Adhesive materials, mechano-adaptive hydrogels, and gradient or layered constructs address different barriers and therefore show different evidence profiles across cartilage-matrix deposition, osteochondral-interface integration, proteoglycan synthesis, chondrogenic differentiation, and bone formation. Symbols encode the apparent directness and convergence of cited evidence; they do not represent effect magnitude, pooled estimates, formal certainty, or rankings. Histological textures are conceptual rather than reproduced or statistically compared data [17,23,44,45,60,61,65,67,68,69,70,71,73,74,75,88,89,90,112,121,128,129,130,131,132,133,134]. The examples in panel C are supported by [17,23,44,45,59,60,61,64,65,66,67,68,69,70,71,72,73,74,75,87,88,89,90,110,112,119,121,125,126,127,128,129,130,131,132,133,134]. The circle symbols use the embedded legend: white, half-filled grey, green, and concentric blue circles denote low, moderate, high, and very high qualitative trends, respectively; a blue horizontal line denotes minimal reported evidence.

**Figure 5:**
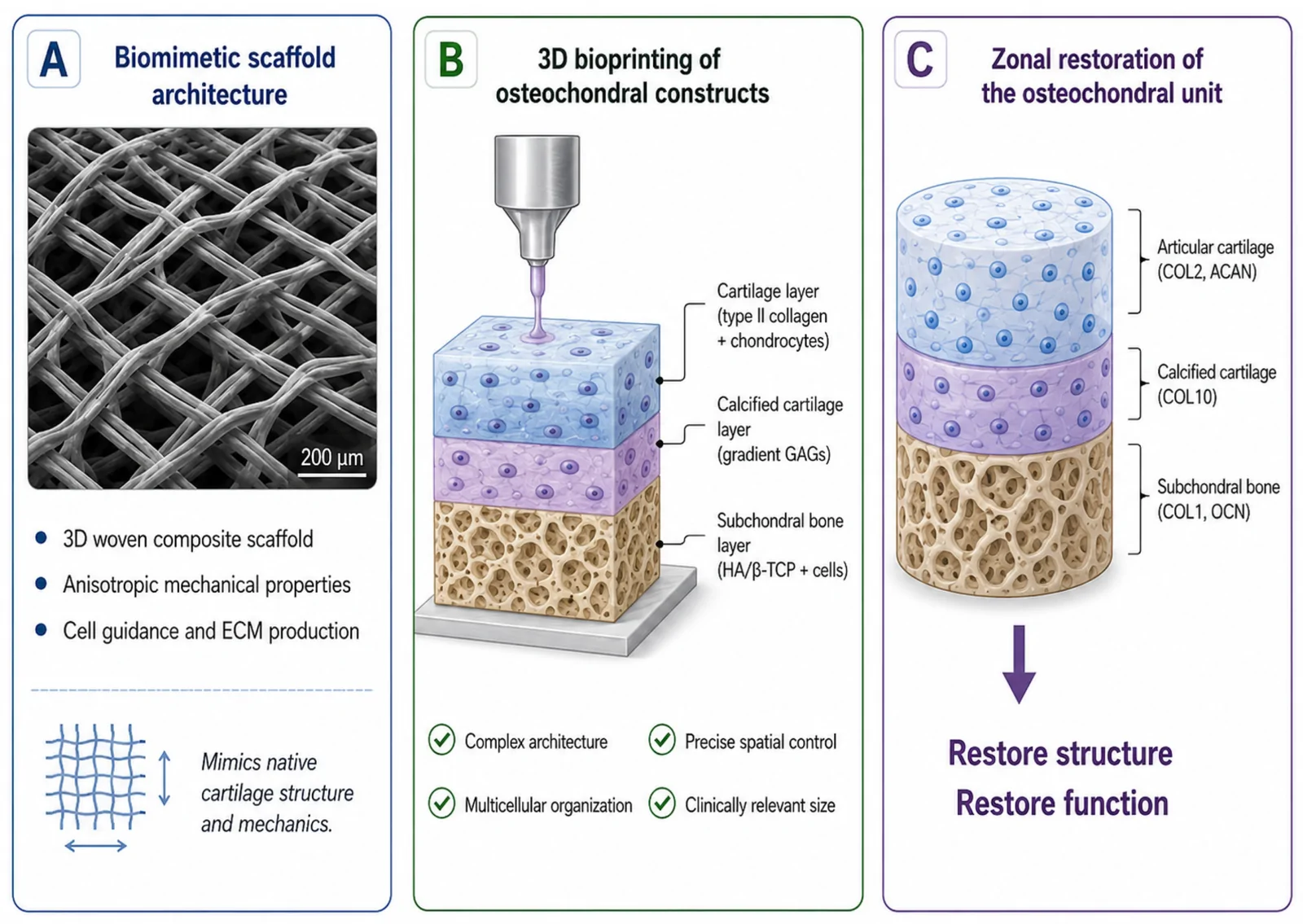
Spatial material design for zonal restoration of the osteochondral unit. Biomimetic woven architectures provide anisotropic mechanical behavior and cell guidance, whereas three-dimensional bioprinting enables layer-specific placement of cartilage, calcified-cartilage, and subchondral-bone components. The intended endpoint is coordinated restoration of articular cartilage, including type II collagen (COL2) and aggrecan (ACAN), the type X collagen (COL10)-rich calcified-cartilage transition, and subchondral bone containing type I collagen (COL1) and osteocalcin (OCN), together with continuous load transfer and interface stability [69,70,71,73,74,75,121,128,129,130,131,132,133,134]. Panel (**A**) shows a woven-scaffold micrograph adapted from Moutos et al. [75]; (**B**) depicts layer-by-layer bioprinting of an osteochondral construct; and (**C**) illustrates the intended zonal restoration. In panel (**A**), double-headed arrows indicate direction-dependent mechanical behavior; the downward arrow in panel (**C**) indicates the intended progression from zonal restoration to structural and functional recovery.

**Figure 6:**
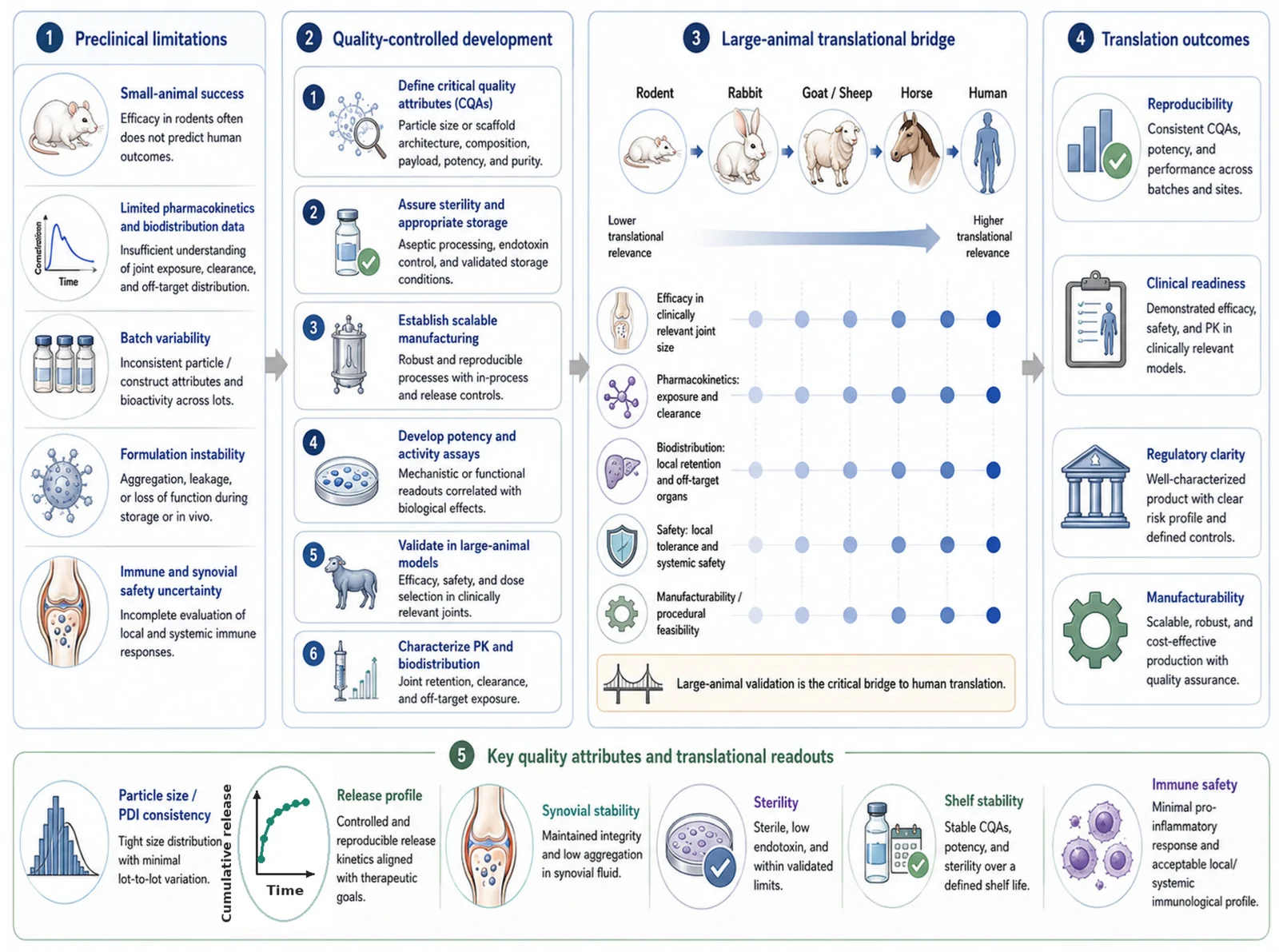
Quality-controlled translational pathway for osteochondral local nanopharmaceutics. Translation requires defining mechanism-linked CQAs, verifying dose delivery and local pharmacokinetics, assuring sterility and storage stability, establishing scalable manufacturing and potency assays, and validating efficacy, fate, and safety in clinically relevant large-animal models. Key outputs are reproducibility, clinical readiness, regulatory clarity, and manufacturability [21,22,77,78,135,136,137,138,139,140,141,142,143,145]. This literature-informed conceptual schematic does not depict original experimental data.

**Table 1:** Conceptual positioning of this review within nano-enabled cartilage and osteochondral repair.

Adjacent Review Domain	Established Contribution	Distinct Pharmaceutical Question Addressed Here
Cartilage-targeted nanoparticles and intra-articular delivery	Defines cartilage targeting, matrix transport, retention, penetration, and charge-mediated interactions [17,18,19,20,23,24,35].	Separates cavity residence, cartilage partitioning, depth-dependent penetration, and cellularly bioavailable cargo.
Nano-enabled hydrogels and local depots	Defines controlled release, network mechanics, degradation, and depot behavior [36].	Introduces mechanopharmaceutics and asks whether nanoscale features causally alter release, mechanics, or cell interaction under loading.
EV-based cartilage repair	Defines EV identity, biological cargo, and reporting standards [37].	Applies a mechanism-necessity test to determine when multicomponent nanobiological cargo is preferable to a defined synthetic payload.
Spatial osteochondral constructs	Emphasizes gradient tissues, multilayered scaffolds, and bioprinting [38,39,40].	Connects nanoscale cue delivery and topography to macroscale cue confinement, interface mechanics, and synchronized degradation.
Nanomaterial translation and fate	Identifies manufacturing, material-identity, and regulatory characterization barriers [21,22].	Distinguishes nominal formulation identity from delivered material state and links CQAs to effective exposure and function.

**Table 2:** Repair barriers, losses of effective exposure or function, nano-enabled responses, and decisive validation.

Barrier	How Exposure or Repair Is Lost	Nano-Enabled Design Response	Decisive Validation
Cavity-to-tissue delivery	Clearance, synovial uptake, and matrix restriction disconnect joint residence from cartilage exposure [28,29,41,42,43].	Reversible matrix binding, tuned size/deformability, local depots, and stage-matched release.	Separate cavity residence, cartilage partitioning, depth distribution, intact-carrier persistence, and active cargo.
Cell-state control	Inflammatory, fibrotic, hypertrophic, or senescent states prevent stable repair [27,50,51,52,53,54,55,56,57,58,59,60,61,62,63,64].	Cargo–carrier combinations, instructive mechanics, controlled degradation, and EVs when coordinated signals are necessary.	Time-resolved immune and stromal states plus anabolic, catabolic, fibrotic, and hypertrophic markers.
Mechanopharmaceutical (emerging evidence)	Cyclic loading may alter depot integrity, fluid exchange, release, friction, and mechanotransduction; direct paired measurements remain sparse [36,47,48,49,67,68].	Viscoelastic, fatigue-resistant, permeable, and tribologically compatible formulations with loading-resilient release demonstrated rather than assumed.	Matched static and loaded release in synovial-fluid-like media with prespecified amplitude, frequency, waveform, and duration; co-measured mechanics, deformation/recovery, mass loss, intact carrier, and active cargo.
Spatial and interfacial	Cartilage, calcified cartilage, and bone require conflicting cues and continuous load transfer [5,69,70,71,72,73,74,75].	Localized nanocarriers, gradients, multilayers, and nanoscale topographies with controlled cue confinement.	Cross-layer leakage, regional matrix and mineral profiles, interface strength, fatigue, and post-degradation continuity.
Delivered material state and translation	Manufacture, storage, protein adsorption, degradation, and dose loss change functional identity [21,22,77,78].	Mechanism-linked CQAs, post-process testing, traceability, and clinically relevant models.	Batch ranges, dose recovery, post-sterilization performance, local/systemic fate, and long-term safety.

**Table 3:** Published quantitative anchors and their use in defining context-specific design windows.

Domain	Published Quantitative Anchor	Decision Use	Boundary or Limitation
Charge-mediated cartilage transport	Avidin: approximately 7 nm, pI ≈ 10.5, K_D ≈ 150 μM and approximately 400-fold uptake versus a neutral, size-matched control [23].	Starting anchor for reversible electrostatic partitioning and depth-resolved exposure.	K_D is not k_off; values depend on carrier valency, ionic/protein environment, matrix charge, and tissue thickness.
Multi-arm avidin delivery	Cartilage uptake approximately 112× bath in healthy tissue and 33× in 50% GAG-depleted tissue; 50% dexamethasone release at 38.5 h and sustained release for approximately 2 weeks [24].	Links disease-dependent partitioning to linker-controlled release and payload exposure.	One carrier–payload family; uptake and release values cannot be transferred directly to other chemistries.
Hydrogel stress relaxation	HA-based formulations: half-stress-relaxation time approximately 60–2000 s at initial modulus near 3 kPa [90].	Starting span for separating relaxation from initial stiffness in chondrogenic screens.	Composition- and cell-dependent; not an acceptance range for load-bearing cartilage repair.
Loading–release coupling	TG-18: 0.5% strain/0.1 Hz versus 35% strain/2.5 Hz, 30 alternating one-minute cycles, with recovery and maintained release [49].	Template for matched static/loaded release plus simultaneous material-state measurement.	Rotational shear, one formulation, and one payload; not a universal physiological compression protocol.
Cross-species cartilage exposure	Avidin half-life approximately 154 h in thick rabbit medial tibial cartilage versus approximately 29 h in rat cartilage; thickness^2^ normalization improved R^2^ from 0.43 to 0.96 [109].	Supports thickness- and tissue-exposure-informed scaling in addition to dose per joint.	Carrier-specific transport model; local exposure cannot be inferred from body-weight scaling alone.

Note: The values in Table 3 are study-specific measurement anchors, not recommended universal cutoffs or batch-release specifications. K_D describes equilibrium affinity and cannot be used to infer k_off without kinetic measurements.

**Table 4:** Nano-enabled modules for cartilage and osteochondral regeneration.

Nano-Enabled Module	Primary Pharmaceutic Function	Principal Failure Risk
Cartilage-targeted and penetrating carriers	Control matrix binding, joint residence, and depth-dependent transport [17,18,84,85].	High binding or positive charge may limit deep penetration and increase irritation.
Responsive carriers	Couple release to inflammatory ROS, pH, or enzyme activity when trigger thresholds are disease-relevant [46].	Trigger thresholds, response kinetics, and off-target activation must be validated in synovial fluid and under mechanical loading.
Nano-enabled and nanocomposite hydrogels	Integrate defect filling, viscoelastic mechanics, cell remodeling, and local release [36,86,87,91,92,93,94,95,96,97].	Reinforcement or filler loading may impair remodeling, create heterogeneity, or promote unwanted mineralization.
Mineral and bioactive phases	Support mineral signaling and bone-facing reconstruction [92,97].	Osteogenic signals must not diffuse into the cartilage layer.
EV nanobiologics	Provide multicomponent cell-state regulation and membrane-mediated interactions [110,111,112,113,114,115,116,117,118,119,120,121,122,123,124,125,126].	Identity, potency, donor-state dependence, storage, and batch consistency must be defined.
Imaging and traceable nanomaterial modules	Map localization and persistence when tracking is integrated with degradation, release, and response measurements.	Signal persistence does not necessarily represent material integrity, cargo activity, or repair.
PLGA-dominant particles versus HA-dominant systems	PLGA emphasizes encapsulation, particle processing, and hydrolysis-controlled release; HA emphasizes matrix interaction, CD44 engagement, hydration lubrication, and injectable networks [80,81,82,83,93,94,95,127].	No universal winner exists; acidic PLGA degradation or burst release and enzymatic HA degradation or cargo escape require payload- and barrier-matched controls.

**Table 5:** Representative studies and their structure–property–exposure implications.

Representative Example	Structure–Property–Exposure Insight	Decision-Critical Unresolved Question
Biofunctional polymer nanoparticles for cartilage targeting and retention [17]	Surface functionality can alter cartilage affinity and joint residence.	What affinity window increases cartilage exposure without surface trapping, synovial sequestration, or loss of reversibility?
Avidin and multi-arm avidin carriers for charge-mediated transport, release, and joint kinetics [23,24,45]	Charge density and multivalency couple binding, penetration, release, and safety.	Which binding–dissociation window remains effective across healthy and degraded matrices?
Cartilage-penetrating nanocarriers for growth-factor delivery [18]	Carrier architecture can improve matrix access and spatial growth-factor distribution.	How do disease stage, glycosaminoglycan loss, and synovial uptake alter penetration?
Extracellular-vesicle and EV-loaded hydrogel systems [110,111,112,118]	EV cargo and adhesive matrices jointly influence cell-state regulation and local retention.	When is coordinated EV cargo mechanistically necessary, and which cell-state potency assay predicts stable repair?
Three-dimensional woven and bioprinted osteochondral constructs [73,74,75]	Architecture and mechanics can be engineered across cartilage–bone regions.	Can zonal cues remain confined while the interface resists fatigue and delamination?
Nanomedicine translation analyses [21,77], regulatory guidance [22], and industry perspectives [78]	Material identity, manufacturing, and post-processing determine whether a mechanism is reproducible.	Which attributes must be controlled because they causally affect in vivo performance?

**Table 6:** Mechanism-linked critical quality attributes and the basis for product-specific acceptance ranges.

Material System	Mechanism-Linked Critical Quality Attributes	Exposure or Clinical Consequence	Basis for Acceptance Range
Nanocarriers	Size distribution, polydispersity index, zeta potential, morphology, surface chemistry, ligand accessibility, deformability, loading, encapsulation efficiency, dose recovery, and release [17,18,85].	Determine cavity persistence, cartilage partitioning, synovial uptake, cellular exposure, safety, and reproducibility.	Establish two-sided limits by linking distribution or surface-state shifts to aggregation, cartilage partitioning/depth, synovial uptake, release, safety, and potency; include method uncertainty and batch capability.
Hydrogels and local depots	Gelation, injectability, viscoelasticity, stress relaxation, permeability, fatigue, degradation, and release [36,48,86,87].	Determine surgical handling, cell remodeling, cyclic-load stability, and local exposure.	Bracket by injection/retention and cyclic-load failure, then narrow using release, cell-remodeling, fatigue, degradation, sterilization, and storage data.
EV-based materials	Cell source, isolation, particle identity, cargo, potency, matrix retention, and storage [37,114,115].	Determine biological consistency and whether complex cargo provides a reproducible mechanism.	Qualify lot-release limits against identity, recovery after matrix incorporation/release, storage stability, and a potency assay correlated with repair-relevant function.
Spatial constructs	Regional composition, gradient continuity, interface strength, cue leakage, fatigue, and degradation [69,73,74,75].	Determine whether zonal architecture becomes stable zonal tissue function.	Set regional limits from cue leakage, interface strength and fatigue, manufacturing resolution, and maintenance after degradation.
Traceable modules	Probe loading, label stability, effects on material properties, and biodistribution [138,139,140].	Ensure that tracking reports mechanism without altering it.	Require unchanged formulation properties and potency, stable label–carrier linkage, and agreement with an orthogonal tissue assay.
Final administered or implanted system	Sterility, storage, post-sterilization mechanics, degradation products, handling, and safety [21,22,78].	Preserve material identity and performance from manufacturing to implantation.	Use process capability and worst-case sterilization, storage, handling, and device-deployment challenges that remain within exposure and functional specifications.

**Table 7:** Context-dependent design variables and failure-oriented validation for cartilage and osteochondral nanomaterials.

Design Variable	Causal Consequence	Required Context-Dependent Measurement	Failure Signal
Particle size, shape, and deformability	Joint retention, synovial uptake, and cartilage transport	Distribution in formulation and synovial-fluid-like media; tissue partitioning; depth-dependent concentration; effective diffusivity [42,43,79,83,84,85]	Cavity retention without cartilage access; aggregation; rapid clearance
Surface charge, ligand density, and matrix affinity	Binding–dissociation balance and protein-corona-dependent targeting	Apparent binding/dissociation, corona composition, synovial uptake, cartilage penetration, and cytotoxicity [17,23,24,44,45,79,84,85]	Surface sequestration, loss of ligand function, or inflammatory uptake
Hydrogel stiffness and viscoelasticity	Cell confinement, mechanotransduction, matrix deposition, and load transfer	Equilibrium/dynamic modulus, stress-relaxation time, creep, and cell-generated remodeling under defined loading [67,68,88,89,90]	Hypertrophy, impaired matrix deposition, extrusion, or insufficient load support
Permeability and degradation	Nutrient transport, cargo release, and replacement by new tissue	Permeability, swelling, mass loss, degradation products, release into synovial fluid, and cyclic loading [36,48,86,87,95]	Burst release, acidic/inflammatory products, or premature mechanical loss
Surface lubrication and wear resistance	Friction, opposing-cartilage protection, and particle generation	Friction coefficient, rehydration, wear volume/debris, and opposing-surface damage under repeated sliding [98,99,100,101]	Friction rise, delamination, debris accumulation, or cartilage abrasion
Spatial architecture and interface connection	Cue confinement, load continuity, and resistance to delamination	Cross-layer leakage, gradient mapping, push-out/shear strength, fatigue, and post-degradation interface testing [134,135,136,137]	Signal mixing, abrupt stress concentration, or interface failure
Host-response profile	Resolution, synovial compatibility, and foreign-body reaction	Time-resolved macrophage/synovial states, blank-material controls, fibrous capsule, and wear-particle uptake [57,58,59,60,61]	Persistent synovitis, fibrosis, or immune activation despite local repair
In vivo fate and material identity	Persistence of the intended structure–property–function relationship	Serial localization, cargo release, chemical transformation, degradation, mechanical retention, and systemic distribution [21,22,77,78,138,139,140,141,142,143]	Label persistence without function, off-target accumulation, or batch-dependent performance
Dose and active exposure	Determines whether retained formulation produces bioavailable cargo in cartilage and target cells	Injected and recovered dose; free versus carrier-associated cargo; cavity concentration; tissue concentration by depth; payload activity; normalization to joint volume or cartilage area [28,29,41,42,43,79,80,81,82,83,84,85]	High total joint signal with low cartilage or cellular exposure; interspecies dose mismatch; loss of activity despite label retention

## Data Availability

No new data were created or analyzed in this study. Data sharing is not applicable to this article.

## Abbreviations

ACAN, aggrecan; AUC, area under the concentration–time curve; CD44, cluster of differentiation 44; CMC, chemistry, manufacturing, and controls; COL1/COL1A1, type I collagen/collagen type I alpha 1 chain; COL2/COL2A1, type II collagen/collagen type II alpha 1 chain; COL10/COL10A1, type X collagen/collagen type X alpha 1 chain; CQA, critical quality attribute; D_eff, effective diffusivity; DLS, dynamic light scattering; ECM, extracellular matrix; EV, extracellular vesicle; FITC, fluorescein isothiocyanate; FOXO, forkhead box O; G′, storage modulus; G″, loss modulus; GAG, glycosaminoglycan; HA, hyaluronic acid; K_D, equilibrium dissociation constant; k_off, dissociation rate constant; k_on, association rate constant; mAv, multi-arm avidin; MISEV, Minimal Information for Studies of Extracellular Vesicles; MMP13, matrix metallopeptidase 13; MRI, magnetic resonance imaging; MSC, mesenchymal stromal cell; OA, osteoarthritis; OCN, osteocalcin; PDI, polydispersity index; PEG, poly(ethylene glycol); PET, positron emission tomography; PIEZO1, piezo-type mechanosensitive ion channel component 1; PK/PD, pharmacokinetics/pharmacodynamics; PLGA, poly(lactide-co-glycolide); pI, isoelectric point; ROS, reactive oxygen species; RUNX2, runt-related transcription factor 2; SOX9/SOX11, SRY-box transcription factor 9/11; TGF-β, transforming growth factor beta; TRPV4, transient receptor potential vanilloid 4.
